# Fungi in Bronchiectasis: A Concise Review

**DOI:** 10.3390/ijms19010142

**Published:** 2018-01-04

**Authors:** Luis Máiz, Rosa Nieto, Rafael Cantón, Elia Gómez G. de la Pedrosa, Miguel Ángel Martinez-García

**Affiliations:** 1 Servicio de Neumología, Unidad de Bronquiectasias y Fibrosis Quística, Hospital Universitario Ramón y Cajal, 28034 Madrid, Spain; luis.maiz@salud.madrid.org (L.M.); rosanr23@hotmail.com (R.N.); 2 Servicio de Microbiología, Hospital Universitario Ramón y Cajal and Instituto Ramón y Cajal de Investigación Sanitaria (IRYCIS), 28034 Madrid, Spain; rafael.canton@salud.madrid.org (R.C.); elia.gomez@gmail.com (E.G.G.d.l.P.); 3 Servicio de Neumología, Hospital Universitario y Politécnico la Fe, 46016 Valencia, Spain

**Keywords:** bronchiectasis, fungi, yeast, filamentous fungi, *Candida albicans*, *Aspergillus*, allergic bronchopulmonary aspergillosis, mycobiome

## Abstract

Although the spectrum of fungal pathology has been studied extensively in immunosuppressed patients, little is known about the epidemiology, risk factors, and management of fungal infections in chronic pulmonary diseases like bronchiectasis. In bronchiectasis patients, deteriorated mucociliary clearance—generally due to prior colonization by bacterial pathogens—and thick mucosity propitiate, the persistence of fungal spores in the respiratory tract. The most prevalent fungi in these patients are *Candida albicans* and *Aspergillus fumigatus*; these are almost always isolated with bacterial pathogens like *Haemophillus influenzae* and *Pseudomonas aeruginosa*, making very difficult to define their clinical significance. Analysis of the mycobiome enables us to detect a greater diversity of microorganisms than with conventional cultures. The results have shown a reduced fungal diversity in most chronic respiratory diseases, and that this finding correlates with poorer lung function. Increased knowledge of both the mycobiome and the complex interactions between the fungal, viral, and bacterial microbiota, including mycobacteria, will further our understanding of the mycobiome’s relationship with the pathogeny of bronchiectasis and the development of innovative therapies to combat it.

## 1. Introduction

Bronchiectasis is defined as chronic inflammatory bronchial disease with irreversible dilation of the bronchial lumen, and it can occur for a number of reasons. Clinically speaking, it usually presents itself with chronic cough and expectoration, and also with recurrent infectious exacerbations. It is mostly accompanied by chronic bacterial infection, as well as isolated yeast and filamentous fungi or molds whose pathogenic role has not yet been clarified [1,2].

The airways are constantly exposed to environmental fungi. The ones that are most commonly isolated in bronchiectasis patients are *Candida albicans* and *Aspergillus* spp. Of these, the various species of *Aspergillus* spp. have the greatest pathogenic potential [3]. The inhalation of fungal spores and conidias has little effect on healthy subjects as their immune mechanisms will function correctly. In chronic pulmonary diseases, such as bronchiectasis, however, fungal growth is enhanced by deteriorated mucociliary clearance, thick mucosity, and the fungis’ capacity to evade the host’s immune mechanisms [3]. Although there are no published data about the real prevalence of co-colonization of fungi and bacteria, in most cases, fungi are isolated with pathogens, such as *Haemophillus influenzae* or *Pseudomonas aeruginosa*, making difficult to determine their pathogenic significance. However, they are associated with a persistent inflammatory response in the airways that can be measured accurately, particularly in those patients with more marked respiratory deterioration.

Microbiological diagnostic techniques for fungal infection have developed enormously in recent years. Although the traditional diagnostic methods (microscopic tests, biochemical analysis, and cultures in selective media) are still used, in many instances they have been superseded by new molecular techniques, such as metagenomic analysis, as well as by the application of mass spectrometry (Matrix-Assisted Laser Desorption/Ionization Time-of-Flight—MALDI TOF) technique [4]. These breakthroughs have made it possible to identify new fungal pathogens, although more studies are needed to determine their clinical significance.

Anatomical modifications, as found in bronchiectasis and immunological alterations, as found in immunosuppressive states, can give rise to a wide range of respiratory fungal diseases, from simple colonizations to invasive aspergillosis or allergic bronchopulmonary aspergillosis (ABPA) [5]. These phenomena are difficult to diagnose in bronchiectasis patients, as their symptoms and radiological presentation are not easily distinguishable from those of the underlying disease. Furthermore, we are still lacking in uniform diagnostic criteria for some of these fungal diseases [6].

## 2. Pathogeny of Bronchiectasis

Bronchiectasis arises as a result of a vicious circle that is produced by bacterial infection and inflammation [7]. The damage to the mucociliary system impedes the elimination of secretions and propitiates the growth of microorganisms, such as bacteria, mycobacteria, and fungi in the airways. Infection and inflammation cause structural bronchial damage and perpetuate this pathogenically vicious circle. An imbalance between pro-inflammatory and anti-inflammatory products and an incomplete resolution of the infection and inflammation, despite treatment and the immune response, could play an important role in the progression of the disease [1]. The role of fungi in this process has yet to be defined.

## 3. Microbiology of Bronchiectasis

The airways of bronchiectasis patients tend to be colonized by potentially pathogenic microorganisms. The bacteria that are most commonly isolated in these patients are *H. influenzae*, *P. aeruginosa*, *Streptococcus pneumoniae*, *Staphylococcus aureus,* and *Moraxella catarrhalis* [8]. Other microorganisms are also often found, such as non-tuberculous mycobacteria (NTM) [9,10], yeasts, and filamentous fungi [11].

In the last ten years, the use of pyrosequencing methods to investigate all the genetic sequences of the microorganisms that are present in the respiratory secretions has revealed an extremely varied bacterial microbiota, which are comparable to that found in other chronic respiratory diseases [12].

## 4. Prevalence of Fungal Infection and Risk Factors

Healthy subjects quickly eliminate fungal conidias via the mucociliary system and then phagocytize them via cells in the immune system. In patients with chronic respiratory diseases, such as bronchiectasis, however, the deterioration of the mucociliary clearance system and thick mucosity allow for these pathogens to persist, and, furthermore, create colonization mechanisms [13,14]. The prevalence of fungal colonization in the airways varies according to the geographical area, the microbiological culturing, and identification methods used and the etiology of the bronchiectasis [15,16]. Any comparison of studies is also complicated by the differing definitions of colonization, infection, and persistence used therein [17,18].

*C. albicans* and *Aspergillus* spp. are the fungi that are most usually isolated in the respiratory secretions of patients with chronic respiratory disease, and they are also the ones that are most often cultured in patients with cystic fibrosis (CF) [19,20] and bronchiectasis [11,21,22]. *Aspergillus fumigatus* is isolated from respiratory secretions of patients with CF in between 9% and 57% of samples, and the rate is somewhat higher in the case of *C. albicans*.

## 5. Yeasts

The yeasts most often found in bronchiectasis belong to the *Candida* spp. genus, with *C. albicans* being the most common species. A recent analysis of the data derived from routine microbiological cultures in various Spanish hospitals showed that *C. albicans* was isolated in 45.2% of the patients that were studied [11]. Other fungal species were recovered from 5.2% of the patients.

It is also not uncommon to identify yeasts in these patients’ respiratory samples. Yeasts such as *Trichosporon beigelli* and *Saccharomyces cerevisiae* form part of the normal microbiota of the upper respiratory tract, digestive system, and even foodstuffs, but their possible implication in the processes of colonization and infection is subject to debate. On the one hand, they could represent contamination derived from the collection of the respiratory sample, but on the other hand, these types of yeast have demonstrated a great immunogenic capacity, which could be linked to the impaired mucociliary clearance found in this group of patients [6].

Finally, the so-called black yeasts have recently been noted in patients with bronchiectasis and in CF. *Exophiala dermatitidis* is particularly prominent in this respect. In some cases in the literature it was the only fungus isolated in patients with a deteriorated respiratory function, and their clinical condition improved after the administration of antifungals and subsequent negative cultures [23].

Furthermore, as in the case of CF, chronic antibiotic treatment may be a major risk factor for respiratory fungal colonization and infection, although more studies are needed to corroborate this hypothesis. One study recently undertaken on bronchiectasis patients found that persistent *C. albicans* was most common in those patients who were receiving chronic antibiotic treatment [11].

## 6. Filamentous Fungi

The various species of the *Aspergillus* spp. genus are the most prevalent filamentous fungi found in bronchiectasis patients (24.2% in the aforementioned multi-centre study) [11]. *A. fumigatus* is the most common species, followed by *Aspergillus niger*, *Aspergillus terreus*, and *Aspegillus flavus*. However, there are huge variations in the prevalence of the fungi isolated in studies, for the reasons noted above. For example, the prevalence of *Aspergillus* spp. in bronchiectasis ranges from 7% to 24% [11,24].

*A. fumigatus* has been the main focus of almost all of the research into fungal diseases, as it is both the most prevalent and the most pathogenic. The other species of *Aspergillus* spp. tend to act more as colonizers than as pathogenic agents. The distribution of the species varies according to the geographical area, with a high prevalence of *A. niger* and *A. terreus* in Japan and of *A. flavus* in India and China [25].

Almost all of the research into the risk factors that are associated with the isolation of *Aspergillus* spp. and other fungi in the respiratory tract has been carried out on patients with CF [18], but even so, its role in this population has still not been totally clarified. It has been suggested that the incidence of *Aspergillus* spp. increases with age [26], with a greater severity of lung disease [26] and with chronic antibiotic treatment [21,27,28].

Although chronic antibiotic treatment may be one of the most significant risk factors for fungal respiratory infection, Máiz et al. did not find any such association with *Aspergillus* spp. Nevertheless, they did link its persistence to greater purulence in the sputum [11]. The lack of this association between *Aspergillus* spp. and antibiotic treatment could be due to the fact the culture of sputum samples may not be the most appropriate way to detect the presence of *Aspergillus* spp. in the lower respiratory tract [29,30,31], although the use of microbiological techniques that are not based on cultures from respiratory samples has been hotly debated in the case of bronchiectasis patients. The detection of the antigen of *Aspergillus* spp. or galactomannan in serum or bronchoalveolar lavage has been proposed as a microbiological criterion for probable invasive aspergillosis in immunocompetent patients with lung diseases, like Chronic obstructive pulmonary disease (COPD) and bronchiectasis, who also fulfil other clinical criteria, such as pulmonary deterioration, frequent exacerbations after antibiotic treatment, or high doses of steroids. These algorithms have only been applied to critical patients, however, further studies are required to validate the detection of galactomannan in immunocompetent patients who do not need to be admitted to an intensive care unit [32,33,34].

Other species of filamentous fungi that have been described in this group of patients include *Scedosporium apiospermum* and various species from the *Fusarium* and *Penicillium* geni. Filamentous fungi from the Mucorales family, such as *Rhizopus* spp. and *Mucor* spp., have also been found. Other species that have recently come to the fore include dematiaceous fungi, such as *Alternaria* spp. and *Bipolaris* spp, whose presence is associated with allergic stimuli of the bronchial airway [35].

Improvements in the methods used for the microbiological identification of filamentous fungi—including the recently introduced MALDI TOF technique—has made it possible to describe “new” species, such as *Geosmithia argillacea*, which is associated with the appearance of exacerbations in CF patients. Conventional identification techniques based on morphological characteristics usually define this as *Penicillium* spp.

Such descriptions of “new” species also extend to so-called cryptic species that are related to *A. fumigatus*: molecular biology techniques have revealed species associated with the Fumigati section, such as *A. ustus*, which would be defined as *A. fumigatus* by morphological identification, but nevertheless has a different profile of sensitivity to voriconazole [36]. Table 1 shows the species that are most frequently found in bronchiectasis patients.

## 7. Pathogenic Mechanisms

Apart from the case of *P. aeruginosa* [39], little is known about the pathogenic role that is played by other bacteria in bronchiectasis, but the role of fungi is even less documented. Most of the studies on the pathogeny of fungi in respiratory diseases have focused on CF and COPD, and the European Academy of Allergy and Clinical Immunology recently drew attention to our lack of knowledge about the connections between fungal infection, the microbiome, and bronchiectasis [40]. So far, any microbiome studies that could cast new light on this matter have placed a particular emphasis on CF [37] and bacterial ecology [41].

The alterations in ciliary clearance and the bronchial tree destruction that occur during the bronchiectasis process, along with the chronic inflammation that is associated with the colonization of the mucosa by various bacterial and viral microorganisms, favour pathogenic colonization by fungi. Moreover, the reduced ciliary clearance in the bronchial tree is particularly relevant in this respect. Whilst healthy people can eliminate most fungal spores or conidias via ciliary clearance—with the remainder being phagocytized by innate immune mechanisms—bronchiectasis patients are unable to eliminate the majority of spores, as the fungal presence is too big for their impaired mucociliary clearance. This means that fungi are retained in the mucosity of their respiratory tree and that, in the case of filamentous fungi, they can invade tissue (colonization-infection processes), cause tissue damage, and provide a stimulus to the humoral immune response [6].

There are three factors that can explain the contribution of fungi to the emergence of bronchiectasis: antigens and fungal proteases; genetic susceptibility; and, interactions that may arise with other microorganisms, such as mycobacteria.

**Figure 1:**
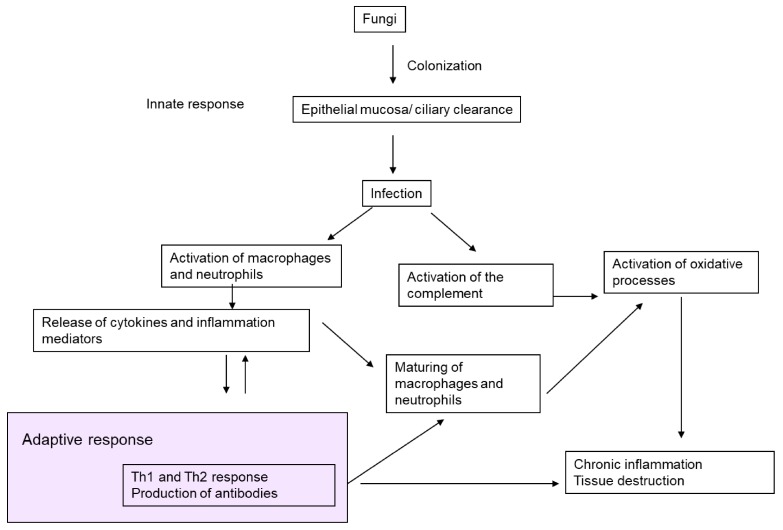
Pathogenic mechanisms of fungi in bronchiectasis.

Both yeasts, and, more particularly, molds have elements in their cellular wall (elastase, collagenase, trypsin, MUC5AC, chitin, β-glucan, gliotoxins) that can degrade the components of the tissue matrix [38,42,43,44,45]. Fungal proteases are produced once the fungus has invaded the mucosa and developed hyphas [46]. Animal models have shown that fungal proteases induce the production of cytokines and other pro-inflammatory mediators [47,48,49]. *Aspergillus* spp. spores are also capable of resisting phagocytic cells [50], neutrophils, and alveolar macrophages [51,52]. This hindrance to the elimination of conidias by the airway macrophages triggers a response in the fungal hyphae, the respiratory epithelial, dendritic and phagocytic cells, and the toll-like receptors [53]. Damage to this line of defence can favour exposure to fungal antigens, producing a Th1-type response in healthy people and a Th2-type response in ABPA patients [54]. Figure 1 presents a schematic summary of the pathogenic mechanisms of fungi in bronchiectasis.

There are various factors that are implicated in the genetic susceptibility to suffer from fungal diseases. For example, in the case of Allergic bronchopulmonary aspergillosis (ABPA), which is unleashed by a Th2 response, an association has been demonstrated with dysfunctions of the cystic fibrosis transmembrane conductance regulator [55], while variations in the prevalence of polymorphisms may explain the differences in the prevalence of ABPA from one geographical area to another.

Apart from fungal proteases and the susceptibility of the host, a third factor in the pathogeny of fungal bronchiectasis is a possible interaction with other microorganisms, such as mycobacteria. It has been demonstrated that Nontuberculous mycobacteria (NTM) can propitiate sensitization to *Aspergillus* and play a major role in the appearance of ABPA in a susceptible host [56].

CF patients have also presented an association between ABPA and isolations of *Aspergillus* and NTM in respiratory samples [57,58]. However, this apparent association could be spurious due to the deterioration of the lung function and the presence of common risk factors for their isolation (e.g., antimicrobial treatment).

## 8. Clinical Significance and Association with Other Microorganisms

The clinical significance of fungal growth in the cultures of respiratory samples that were taken from bronchiectasis patients has not been clearly established because, on the one hand, few studies have examined its epidemiology, and, on the other, the criteria usually applied to the definition of chronic colonization are adapted from definitions of chronic colonization by other microorganisms, such as *P. aeruginosa* [6]. The issue of clinical significance is complicated still further by the fact that fungi are not usually isolated on their own. More than one fungal species may be found (the most common being *A. fumigatus* and *C. albicans*) and they are often accompanied by other types of microorganisms, such as bacteria.

Apart from the aforementioned association with NTM [59], the model of the relationship with chronic bronchial infection described in the literature is very similar to that of CF patients, since there is a relationship between the appearance of filamentous fungi (especially *A. fumigatus*) and chronic bronchial infection by *P. aeruginosa.* No fungi are isolated, however, in the cultures of patients with chronic colonization by *H. influenzae* [11]. Finally, the possible role of respiratory viruses in bronchial inflammatory processes needs to be mentioned, as these could favour colonization by other types of microorganisms, including bacteria and fungi.

## 9. Clinical Spectrum of *Aspergillus*

**Figure 2:**
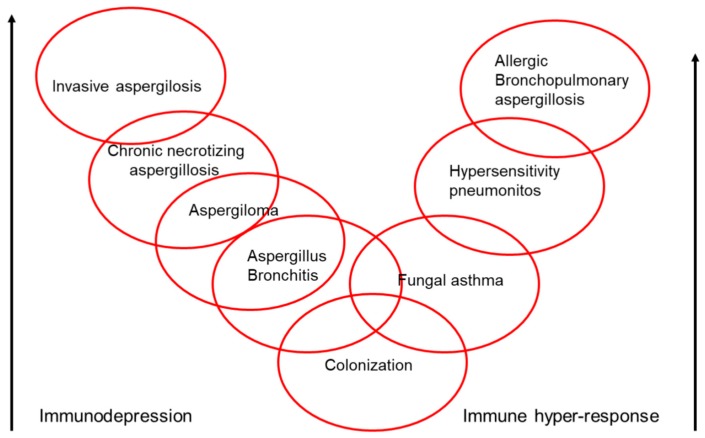
Pathogenic potential of *Aspergillus* spp. (arrow direction means more severity).

Various species of *Aspergillus* can colonize the airways without any pathogenic consequences, but they are also capable of causing several types of disease: bronchitis due to *Aspergillus* spp., aspergilloma, chronic necrotizing aspergillosis, invasive aspergillosis, and asthmatic reactions (bronchial asthma, extrinsic allergic alveolitis, and ABPA) (Figure 2). *Aspergillus* spp. can create several different clinical pictures at the same time in a single patient, and these can evolve over time in accordance with the progression of the underlying pathology and the patient’s immunity. All of this makes it even more difficult to diagnose and treat fungal disease in bronchiectasis patients. Of all the clinical pictures that are produced by fungi, the one most often associated with bronchiectasis is allergic bronchopulmonary mycosis.

## 10. Allergic Bronchopulmonary Mycosis

Allergic bronchopulmonary mycosis is responsible for persistent asthmatic symptoms, pulmonary eosinophilia, radiological infiltrates, and proximal bronchiectasis. There are many fungi that are capable of triggering it [60,61], but *A. fumigatus* is behind more than 90% of cases, giving rise to what is known as ABPA. The manifestations of this group of diseases are affected both by the virulence of the fungus in question and by the patient’s atopic immunological response. The prevalence of ABPA in idiopathic bronchiectasis is 10% [62]. *A. terreus* has been implicated in approximately 10% of patients with ABPA in Japan [63,64], but this species is not very pathogenic and is rarely associated with other types of fungal disease in bronchiectasis. *A. nidulans* has also been described as a cause of ABPA in bronchiectasis patients [65].

ABPA rarely causes bronchiectasis [66]. Both the European [2] and Spanish guidelines [1] for bronchiectasis recommend ruling out ABPA in all of the patients diagnosed with bronchiectasis, by screening by means of a total IgE serum test and specific IgE and IgG tests for *A. fumigatus*, or alternatively a prick test for *A. fumigatus* [2]. These recommendations are often ignored, however, as evidenced by a review recently undertaken in the United Kingdom [67].

It is difficult to diagnose ABPA in a bronchiectasis patient as both diseases share many clinical and radiological characteristics. An attempt to unify the diagnostic criteria for ABPA has recently been made in a review carried out by a working group from the International Society for Human and Animal Mycology [55].

ABPA intensifies the deterioration in the lung function and increases the number of exacerbations in both CF and bronchiectasis [68,69], but it is still not known whether the presence of *A. fumigatus* and the triggering of ABPA are a cause or consequence of pulmonary deterioration. The mechanisms by which sensitization to *A. fumigatus* gives rise to ABPA, and by which ABPA in its turn triggers bronchiectasis, have not been clearly established, but it has been speculated that they could be the result of remodelling of the airways subsequent to the inflammation that is caused by continuous exposure to *A. fumigatus* [38].

Although ABPA is associated, in both CF and bronchiectasis, with a greater prevalence of airway infection by other pathogenic microorganisms, such as NTM [56,57], it is not known whether this association is due to defects in the cystic fibrosis transmembrane conductance regulator that could occur in patients with bronchiectasis, or whether this explanation is only applicable to CF patients [70].

## 11. Mycobiome

The recent application of mass sequencing techniques with high-performance platforms and amplification of total DNA in the respiratory secretions of patients with chronic bronchopulmonary disease has made it possible to detect a wider range of microorganisms than was the case with conventional cultures [71]. Apart from classic bacteria like *P. aeruginosa*, *H. influenzae*, *S. pneumoniae*, and *S. aureus*, bacteria that grow exclusively in anaerobiosis have been found, along with molds and yeast; these are known collectively as the mycobiome [72]. Differences have been observed between the yeast identified in healthy individuals and those detected in patients with bronchiectasis or asthma [73], suggesting that they could have a different significance in different contexts.

Most of the research undertaken on the microbiome in respiratory disease has involved small sample sizes, as well as sputum samples (rather than bronchoaspiration), and as a result, its clinical importance is still unclear. The main focus has been on the bacterial microbiome in CF, asthma, and COPD [74,75,76,77], with only a few studies of bronchiectasis [12]. In the case of CF, fungi are detected more often during exacerbations, and, in many cases, after antimicrobial treatment, although one follow-up study of six patients who gave a series of respiratory samples and had their complete microbiome analyzed presented a relative stability of the various fungal species found, including *Candida* spp. [73].

Studies of the microbiome have demonstrated that resistance increases and bacterial diversity decreases after antibiotic treatment, although this effect disappears a few weeks after the completion of treatment, with a subsequent recovery of the previous microbial composition [77].

**Figure 3:**
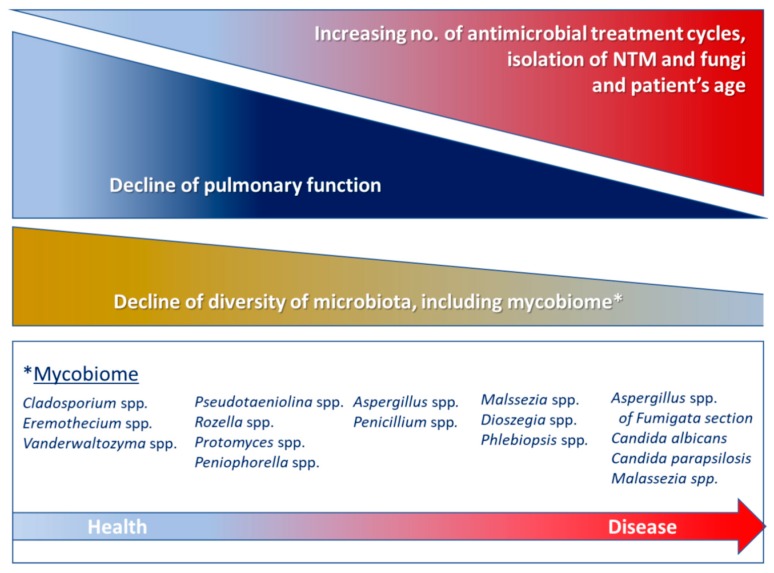
Evolution of the mycobiome in patients with chronic respiratory disease. Information has been obtained from reference [72].

Apart from the infections that are already known to be caused by fungi, the lung mycobiome can have inflammatory effects that can cause or aggravate chronic respiratory disease. Like bacteria, fungi also contain pathogen-associated molecular patterns (PAMP), which are recognizable by pattern recognition receptors that activate immune cells (macrophages, B and T cells), thereby triggering inflammation. Given that fungi are omnipresent in the environment, the respiratory system’s continuous exposure to them and their capacity to trigger an inflammatory process means that the mycobiome may contribute to lung damage, which makes it important to analyze it to establish its real pathogenic role [78]. Most studies have found that, as in the case of bacteria, a reduction in fungal diversity in respiratory diseases correlates with a poorer lung function (along the same lines as a reduction in bacterial diversity). This reduced diversity could be caused by the excessive growth of one fungal species or by the elimination of other species. Figure 3 shows the evolution of the mycobiome in patients with chronic respiratory disease [72].

There are several justifications for research into the lung mycobiome in patients with bronchiectasis. Firstly, the respiratory tract constitutes the main point of entry for fungal spores; secondly, it has been demonstrated that bronchiectasis is a risk factor for fungal disease; and, thirdly, fungi can exacerbate the respiratory deterioration of such patients. Increased knowledge of the complex interactions between the fungal, viral and bacterial microbiota would probably propitiate the development and implementation of innovative therapies. Improvements in sequencing techniques and their interpretation (as well as a reduction in their cost) could be useful in furthering our understanding of the microbiome in both healthy and sick people. Furthermore, research into the relationship between the digestive and respiratory microbiota could also provide insight into the pathogenesis of bronchiectasis.

## 12. Future Research

There are still many tasks pending with respect to fungi and bronchiectasis, such as, for example, establishing their prevalence and the best method for detecting them; assessing the pathogenic significance of individual species; verifying whether fungi are a cause or consequence of bronchiectasis; defining the risk factors for fungal infection and the importance of sensitization to fungi; and, evaluating tests to diagnose invasive aspergillosis in bronchiectasis patients and to detect interactions between the mycobiome and the host.

In a document recently published by the EMBARC Clinical Research Collaboration, Aliberti set out the following research priorities [79]:

1. Studies on bronchiectasis patients in a stable phase and in exacerbations, to evaluate how they are affected by fungi.
2. Studies on the microbiome (including bacteria and potentially pathogenic fungi) with detailed data on phenotypical studies.
3. Studies on the prevalence of fungi.
4. Studies on fungi (both alone and in joint infection with other pathogens) in bronchiectasis patients in a stable phase and in exacerbations, and on the impact of fungi on the severity and evolution of the disease in these patients.
5. Studies to evaluate whether chronic antibiotics are a predisposing risk factor for fungal respiratory diseases.

## 13. Conclusions

The growth of fungi in the conventional microbiological cultures of respiratory samples from bronchiectasis patients has been linked to the triggering of inflammatory response in the bronchi. The species that are most commonly found belong to the genera *Aspergillus* spp. and *Candida* spp.

Although we do not know the risk factors for fungal colonization and infection in bronchiectasis, chronic antibiotic treatment is one of the most important risk factors. The clinical significance of the growth of fungi in cultures of respiratory samples taken from bronchiectasis patients has still not been clearly defined. Further studies that would make it possible to standardize and evaluate both the microbiological criteria for defining chronic colonization and the methods used for culturing and identifying fungi would provide us with more precise knowledge of the genera and species involved, and of the role that fungi play in the clinical evolution of bronchiectasis patients.

## Figures and Tables

**Table 1:** Species most frequently found in patients with bronchiectasis not associated with cystic fibrosis [6,11,21,23,37,38].

Yeasts	Filamentous Fungi
*Candida albicans*	*Aspergllus fumigatus*
*Candida glabrata*	*Aspergillus niger*
*Candida parapsilosis*	*Aspergullus terreus*
*Saccharomyces cerevisiae*	*Scedosporium apiospermum*
*Trichosporon beigellii*	*Penicillium* spp.
*Exophiala dermatitidis*	*Fusarium* spp.
